# Asymptomatic sensitization to a cow’s milk protein induces sustained neuroinflammation and behavioral changes with chronic allergen exposure

**DOI:** 10.3389/falgy.2022.870628

**Published:** 2022-09-07

**Authors:** Afrina Brishti, Danielle L. Germundson-Hermanson, Nicholas A. Smith, Angela E. Kearney, Yassmine Warda, Kumi Nagamoto-Combs

**Affiliations:** ^1^ Department of Biomedical Sciences, University of North Dakota School of Medicine & Health Sciences, Grand Forks, ND, United States; ^2^ Clinical and Translational Science Graduate Program, University of North Dakota School of Medicine & Health Sciences, Grand Forks, ND, United States

**Keywords:** beta-lactoglobulin, food allergy, desensitization, immunoglobulin, cytokine, chemokine, GFAP, Iba-1

## Abstract

Mouse models of food allergy have contributed to our understanding of various aspects of the disease, including susceptibilities, symptom spectra, cellular mechanisms, and therapeutic approaches. Previously, we used a mouse model of non-anaphylactic cow’s milk allergy (CMA) and investigated sex- and strain-dependent differences in immunological, neurological, and behavioral sequelae. We showed that male C57BL/6J mice sensitized to a bovine whey protein, β-lactoglobulin (BLG; Bos d 5), exhibited anxiety- and depression-like behavior upon acute allergen challenge. Systemic levels of BLG-specific immunoglobulins, cytokines and chemokines were also elevated in the sensitized mice. Furthermore, neuroinflammation and intestinal dysbiosis were evident as the possible causes of the altered behavior. To assess whether frequent allergen exposure influences CMA-associated pathologies over an extended period in this subclinical model, we placed BLG-sensitized mice on a whey protein (WP)-containing or whey-free control (CTL) diet for 3 months. As expected, allergen-specific IgE was significantly elevated in the plasma after completing the 5-week sensitization phase. However, the IgE levels declined in both diet groups after 3 months. In contrast, allergen-specific IgG1 stayed elevated in sensitized mice with the CTL diet, and the WP diet to a lesser extent. Interestingly, BLG-sensitized mice on the WP diet exhibited anxiety-like behavior and a trend toward spatial memory decline compared to the sham or the sensitized mice on the CTL diet. Moreover, increased immunoreactivities for GFAP and Iba1 and elevated levels of CXCL13 and CCL12, the chemokines involved in central leukocyte recruitment and other neurological diseases, were also observed in the brain. We demonstrated that sensitization to the whey protein, particularly with continuous allergen exposure, resulted in persistent neuroinflammation and associated behavioral changes despite lowered allergen-specific immunoglobulin levels. These results suggested that continuous consumption of the offending allergen may lead to adverse consequences in the brain even after desensitization.

## Introduction

Food allergy has become progressively common worldwide (1). In the United States alone, over 10% of its population is estimated to be afflicted (2, 3). Numerous efforts are continuously made to broaden our understanding of disease development, predispositions, and therapeutic strategies both at clinical and preclinical levels. However, individual differences in offending allergens, symptom presentations, and comorbidities with other atopic diseases among human subjects pose challenges to the standardization of patient selection and stratification across clinical studies. Furthermore, inconsistencies in other factors, such as genetic background, diet, and living environment, can also introduce variables that potentially affect study outcomes (4–6). Thus, the use of animal models in controlled laboratory environments mitigates these challenges by allowing investigators to regulate potential internal and external factors. Various animal models of food allergies have been established thus far and contributed to the current knowledge in the field (7, 8).

In particular, mouse models of food allergies have been widely used to investigate different aspects of the disease, including but not limited to the mechanism of hypersensitivity (9), strain differences in immune responses (10, 11), routes of sensitization (12–15), characteristics of allergenic substances (15, 16), and efficacies of therapeutic approaches (10, 17). Typically, the sensitization regimens used in these studies aim to cause robust hypersensitivity in mice, and acutely challenging sensitized mice results in anaphylaxis. These models closely reproduce the symptoms of patients with severe allergic reactions and thus, are valuable for assessing the effects of experimental variables on the extent of symptom manifestations.

Importantly, some individuals have an immune phenotype known as asymptomatic sensitization or sensitized tolerance. This group exhibits elevated levels of allergen-specific IgE despite the lack of physical reactions to the allergens (18, 19). Unlike those with severe allergic reactions, such asymptomatically sensitized individuals tolerate the consumption of offending allergens well and do not require strict allergen avoidance. However, recent studies that differentiated symptomatic and asymptomatic patients demonstrated that the immune cells from the latter group showed distinct immune activation upon allergen exposure compared to both allergic and non-allergic groups (19, 20). These observations not only underscore immune system dysfunction in asymptomatic patients but also suggest that immune activation occurs when allergens are consumed even in the absence of physical reactions. Unfortunately, limited information on the pathophysiology of asymptomatic sensitization is available because allergen-sensitized patients without reactions to oral allergen challenges are excluded from many clinical studies and thus understudied.

While many food allergies can cause life-threatening systemic reactions, it has been reported that individuals with cow’s milk and hen’s eggs allergies are more likely to experience less severe symptoms (21). Thus, we previously developed a mouse model of non-anaphylactic cow’s milk allergy (CMA) to investigate the sequelae of asymptomatic sensitization. In this model, C57BL/6J mice were sensitized to bovine whey protein (WP) isolate or purified β-lactoglobulin (BLG; Bos d 5). Although WP- or BLG-sensitized mice did not show severe clinical signs of anaphylaxis, including respiratory distress, lack of movement or death after acute allergen challenge, their circulating allergen-specific IgE levels were significantly elevated (11, 22–24). Interestingly, the sensitized male mice, but not female mice, showed anxiety- and depression-like behavior when assessed 1–2 days after the challenge. Moreover, this behavioral phenotype was also associated with elevated serum levels of inflammatory cytokines, neuroinflammatory pathologies, and intestinal dysbiosis in the sensitized mice (11, 22–24). These results demonstrated that immunological, intestinal, neurological, and behavioral abnormalities develop in asymptomatically sensitized mice, indicating that this subtype of allergic sensitization warrants further investigation.

In the present study, we sought to determine whether repeated consumption of allergen-containing food (i.e., the failure to avoid offending allergens) would have lasting effects in asymptomatically sensitized individuals by sustaining low-grade inflammation. We examined adverse outcomes of an allergen-containing diet after 3 months in asymptomatically sensitized male C57BL/6J mice, including their CMA-associated behavioral changes, immune responses, and neuroinflammation.

## Materials and methods

### Animals

Three-week-old male C57BL/6J mice were purchased from the Jackson Laboratory (Bar Harbor, ME, USA) and housed in a specific pathogen-free facility with a 12-h light/12-h dark cycle at the University of North Dakota. Mice were housed at 4 animals per cage and acclimated for one week with *ad libitum* access to a whey-free rodent diet (Teklad 2018, Envigo, Indianapolis, IN, USA) and ultra-filtered water. Prior to the experiments, mice were randomly divided into either sham or BLG-sensitization groups (24 mice per group). Each of these two groups was further subdivided into groups of 12 mice that received either a whey-free control diet (CTL) or a whey-protein-containing diet (WP) during the allergen exposure phase. All experimental procedures using mice were approved by the University of North Dakota Institutional Animal Care and Use Committee (IACUC).

### Sensitization and post-sensitization allergen exposure

**Figure 1:**
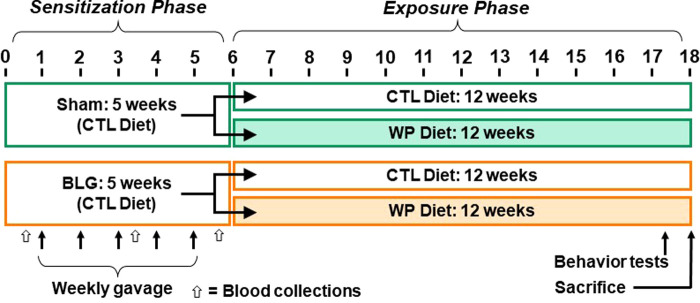
Experimental timeline. Four-week-old male C57BL/6J mice were subjected to a 5-week sensitization regimen. Starting at Week 1, the BLG sensitization groups received weekly oral administration of 1 mg BLG in 200 μl of bicarbonate buffer with 10 µg cholera toxin as an adjuvant, while the sham groups received only the adjuvant in the buffer (arrows). Upon completion of the sensitization phase, mice were placed on either a whey-free control (CTL) or 0.3% whey protein (WP) diet from Week 6 for additional 12 weeks. During the last week of the exposure phase, behavior tests were performed. The numbers indicate experimental weeks. The black, thin arrows indicate the timing of 5 weekly gavages, and the open arrows indicate the timing of blood collection.

A weekly sensitization was performed as previously described (11, 25). Briefly, sham mice received 200 µl of carbonate-buffered vehicle (0.2 M sodium carbonate/bicarbonate [pH 9.0]) containing 10 µg cholera toxin (CT; Cat# 100B, List Biological Laboratories, Inc., Campbell, CA, USA), while the BLG group received 1 mg of BLG (Cat# L0130 MilliporeSigma, Burlington, MA, USA) in the vehicle with CT. The sham or BLG solution was given to each mouse once a week for 5 consecutive weeks *via* intragastric gavage using a soft-tip feeding tube attached to a 1-mL syringe. Upon completion of the sensitization regimen, each of the sham and BLG-sensitized groups was subdivided into two groups (*n* = 12 per group) so that one-half of the groups remained on the whey-free control diet (CTL) while the other half was placed on an allergen-containing diet with 0.3% whey protein (WP; Teklad 8,640 Envigo). This method of continuous diet-derived allergen exposure has been shown to significantly increase BLG-specific IgE levels after 2 weeks (25). The mice were fed the assigned diet for 3 months (12 weeks) until they were sacrificed. A schematic representing the sensitization, post-sensitization allergen exposure protocol and experimental groups is shown in Figure 1.

### Behavioral assessments

#### Open field test

During the 12th week of the allergen exposure phase (Figure 1), a series of behavior tests were performed with the sham and BLG groups that received CTL or WP diet. First, the open field test (OFT) was conducted as described previously (11) to assess the overall activity of mice to explore the 40.6 cm × 40.6 cm × 38.1 cm enclosure (San Diego Instruments, San Diego, CA, USA) for 10 min after 30 s of acclimation. The frequency and time spent in the 20 cm × 20 cm center zone were also measured as an indicator of anxiety-like behavior, signifying the avoidance of the open, unprotected area of the enclosure. The movements of each mouse were recorded with a CCD digital video camera placed above the enclosure (C525 HD webcam, Logitech International, Newark, CA, USA), and the behavioral parameters were computed using the ANY-maze software (Stoelting Co., Wood Dale, IL, USA).

#### Cross maze test

Spatial working memory was assessed using the cross maze test (CMT) as previously described (11, 26). In brief, mice were individually placed in one of the four identical arms of the cross-shaped, white plexiglass maze and allowed to explore the maze for 12 min without additional external stimuli. The movement of mice was recorded using a CCD digital video camera placed above the maze (Logitech International). The entries into the four arms of the maze were manually quantified by two observers who were blinded to the experimental condition of the mice. An “entry” into an arm was counted when all four paws were placed in the opening to the arm. A “successful alternation” was defined as a set of consecutive entries into all of the 4 arms without entering into the same arms twice and expressed as “percent (%) alternations” calculated using the following formula:

$$\%\;\mathrm{alternations}=\frac{\mathrm{number}\;\mathrm{of}\;\mathrm{alternations}}{\left(\mathrm{total}\;\mathrm{entries}-3\right)}\times 100$$

#### Elevated zero maze

The elevated zero maze (EZM) was used as an additional approach to evaluate anxiety-like behavior (11, 26). Mice were initially placed in one of the two walled zones of the 30 cm tall EZM apparatus (Stoelting Co.), and their activities were recorded for 10 min with an overhead CCD digital video camera (Logitech International). The latency and frequency of entries to the open zones and the time spent in the open zones were quantified using the ANY-maze software. The computed results were manually confirmed by observing the recorded videos by a blinded experimenter.

### Biological sample collection

Blood samples from mice were collected from tail veins before (Week 0), during (Week 3), and shortly after the completion of the sensitization procedure (Week 5). Atrial blood was also collected at the time of sacrifice after euthanizing the mice *via* CO_2_ asphyxiation. The tail vein blood and part of the terminal blood were collected in EDTA-coated Microvette tubes (Sarstedt, Inc., Newton, NC, USA) to prepare plasma samples by centrifuging at 2,000 x *g* for 15 min. The remaining terminal blood was used to prepare serum samples by allowing the blood to coagulate for 30 min and centrifuging at 2,000 x *g* for 15 min. Brains and intestines were collected after transcardiac perfusion with phosphate-buffered saline (PBS, pH 7.4). Brains were bisected sagittally. The left hemispheres were immersed in 4% paraformaldehyde for 24 h at 4 °C for histological analyses. The right hemispheres were further dissected into 8 regions as described previously (23), flash-frozen in liquid nitrogen, and stored at −80 °C until used for biochemical analysis. Ileum segments of the intestines were immersion-fixed in Carnoy's solution containing 60% (v/v) absolute ethanol, 30% (v/v) chloroform, and 10% (v/v) glacial acetic acid for 24 h at 4 °C. The fixed tissues were subsequently stored in 70% ethanol at 4 °C until embedded in paraffin for the preparation of histological sections.

### Detection of BLG-specific immunoglobulins

BLG-specific IgE and IgG1 in the plasma samples were quantified by enzyme-linked immunosorbent assays (ELISA) as previously described (11, 27). Briefly, 96-well ELISA plates were coated with 2 mg/ml BLG in 100 mM sodium carbonate/bicarbonate buffer (pH 9.5) overnight at 4 °C. Plasma samples were first diluted to 1:40, and IgG was adsorbed with magnetic protein G-beads (ThermoFisher Scientific, Waltham, MA, USA) according to the manufacturer’s instruction. The resulting supernatants were placed in the BLG-coated wells blocked with an assay buffer (PBS containing 0.5% bovine serum albumin and 0.05% Tween-20). For IgG1 detection, the total IgG adsorbed by the magnetic beads was eluted with 50 mM glycine (pH 2.8) and neutralized with 1 M Tris buffer (pH 7.5), and the eluates were placed in BLG-coated wells. After overnight incubation at 4 °C, the samples were removed from the wells, and the amounts of BLG-specific IgE and IgG1 were quantified using biotinylated anti-mouse IgE (used at 1:1,000, Thermo Fisher Scientific Cat# 13-5992-82, RRID: AB 466,857) and IgG1 (used at 1:1,000; ThermoFisher Scientific Cat# 13-4015-82, RRID: AB 11,220,482), respectively, with avidin-HRP (used at 1:500; ThermoFisher Scientific; Cat# 18-4100-51) and TMB (3,3′,5,5′-tetramethylbenzidine, ThermoFisher Scientific). The enzymatic reactions were terminated with 2 N sulfuric acid, and the plates were read at 450 nm on a BioTek ELx 800 microplate reader with Gen5 software with a reference wavelength at 550 nm (BioTek Instruments, Winooski, VT, USA).

### Quantification of cytokines and chemokines

The levels of mast cell protease 1 (MCPT-1) and CXCL13 (also known as B-lymphocyte chemoattractant, BLC) in the serum were quantified using the Mouse MCPT-1 Uncoated ELISA Kit (Thermo Fisher Scientific, Cat# 88-7503) and CXCL13 DuoSet® ELISA Kit (R&D System Inc., Minneapolis, MN, USA; Cat# DY470) according to the protocols provided by the manufacturers. In brief, 96-well ELISA plates supplied with the respective kits were coated with the capture antibody overnight as directed in the protocol. Six μl of each serum sample was diluted with sample diluent up to a total volume of 100 μl and added to the well following washing and blocking. The plates were incubated overnight at 4 °C. The analytes in the samples were visualized by subsequent incubation with the detection antibody, the streptavidin-HRP and TMB (3,3′,5,5′-tetramethylbenzidine). The enzymatic reactions were terminated with 2 N sulfuric acid, and the plates were read at 450 nm, as described above. The concentrations of analytes were calculated from the respective standard curves prepared according to the manufacturer’s instructions.

CXCL13 and chemokine ligand 12 (CCL12) in hippocampal brain lysates were quantified using the CXCL13 (R&D System Inc.; Cat# DY470) and CCL12 (R&D Systems, Cat# DY428) DuoSet® ELISA Kits. The hippocampal tissues dissected from the right hemispheres were lysed in RIPA buffer (20 mM Tris, pH 7.4, 150 mM NaCl, 1 mM Na_3_VO_4_, 10 mM sodium fluoride, 1 mM EDTA, 1 mM EGTA, 0.2 mM phenyl-methyl-sulfonyl-fluoride, 1% Triton X-100, 0.1% SDS, 0.5% deoxycholate) supplemented with protease inhibitor cocktail (Sigma-Aldrich, Cat# P8340). Protein concentrations of the hippocampal lysates were determined using the Bradford assay (28), and 10 µg of total proteins were used for the ELISA. The analytes in the samples were detected according to the procedure described above.

### Immunohistochemistry

#### Brain tissue sections

The paraformaldehyde-fixed brain tissues were frozen-sectioned at 40 µm as previously described (29) and stored in a cryoprotective solution containing 30% (v/v) ethylene glycol and 30% (w/v) sucrose in 0.1 M phosphate buffer (pH 7.4) at −20 °C until use. Prior to immunohistochemical staining, brain sections were thoroughly rinsed in PBS and treated with 0.3% H_2_O_2_ for 10 min to reduce endogenous peroxide activity. The sections were blocked with PBS containing 0.5% bovine serum albumin and 5% normal goat serum for 1 h and incubated with anti-GFAP (used at 1:1,000, Cell Signaling Technology, Cat# 12,389, RRID: AB_2631098), anti-Iba1 (used at 1:1,000, FUJIFILM Wako Chemicals USA, Corporation, Richmond, VA, USA; Cat# 019-19,741, RRID: AB 839,504), or anti-CD45 (used at 1:500, BioLegend, San Diego, CA, USA; Cat# 103,102, RRID: AB 312,967) diluted in the same buffer. After overnight incubation with primary antibodies at 4 °C, the sections were thoroughly rinsed before target visualization using host-specific secondary antibodies and Vector Elite ABC kit (Vector Laboratories, Burlingame, CA, USA; Cat# PK-6101). Vector VIP substrate kit was used as the chromogen (Vector Laboratories; Cat# SK-4600).

#### Intestinal tissue sections

The intestinal tissues fixed in Carnoy's solution were embedded in paraffin, sectioned at 10 µm, and mounted on glass slides. Before staining, the slides were incubated at 60 °C for 30 min and deparaffinized in Histo-Clear II (National Diagnostics, Atlanta, GA, USA). Tissues were rehydrated through ethanol gradients prior to staining with anti-CD45 antibody (used at 1:500, BioLegend; Cat# 103,102, RRID: AB 312,967). Visualization of the target antigens was performed as described for the brain sections above. The stained sections were analyzed by a pathology resident (YW), who was blinded to the identifications of the tissue samples or the experimental conditions of the animals. The density of the immunoreactive cells and the robustness of the staining were qualitatively evaluated, compared among the experimental groups, and ranked by the staining intensity.

### Quantitative analyses of immunohistochemical staining

Whole-slide images were captured using the Hamamatsu NanoZoomer 2.0HT Brightfield + Fluorescence Slide Scanning System (Hamamatsu Photonics, Bridgewater, NJ, USA). Exposure settings were kept consistent for comparison. The image files (.ndp) were imported into QuPath v3.2 image analysis software (30) to determine the optical density (OD) or percentage of stained cells for quantitative comparisons of immunohistochemical staining. For GFAP and Iba1 immunostaining, an analysis of relative OD was performed for each treatment group. First, the background was normalized between slides by applying the same staining vectors. Then the OD values of the staining were obtained from at least 4 sections containing the hippocampus for each animal, 960 µm apart. The hippocampus was defined as the region of interest (ROI), and Simple Linear Iterative Clustering (SLIC) superpixel analysis was used to separate the ROI into “tiles”, from which the mean OD was calculated relative to the area of the ROI. To analyze the CD45 staining from the brain and intestine sections, the percentage of immunopositive cells was calculated using QuPath. At least 3 fimbria-containing sections were selected, 960 µm apart. In the intestine, 3 ileum sections from each animal were used as the ROIs. The pixel classifier Random Trees (RTrees) was trained to identify the CD45 staining from the brain and ileum sections. Upon successful training of RTrees, the pixel classifier was applied to the ROIs and expressed as the percentage of positive cells.

### Statistical analysis

Differences between experimental groups were statistically analyzed using GraphPad Prism v9.0 software (GraphPad Software, Inc., La Jolla, CA, USA). Student’s t-test was used to calculate *p*-values when differences between the sham and BLG-sensitized groups were directly compared. For the comparison of two groups that did not have the same sample numbers or a normal distribution, Mann-Whitney’s *U*-test was employed. Two-way ANOVA was used with Tukey *post hoc* test when assessing the effects of sensitization status and diet type as the variables. Where indicated, Fisher’s least significant difference (LSD) test was performed with two-way ANOVA to compare the specific effect of one of the variables among the groups. The ROUT method (Q = 1%) was used to identify outliers and removed from the final analysis where indicated. A *p*-value less than 0.05 (*p* < 0.05) was considered statistically significant.

## Results

### Sensitization-induced allergen-specific immunoglobulins in the plasma declined after the long-term consumption of the WP diet

**Figure 2:**
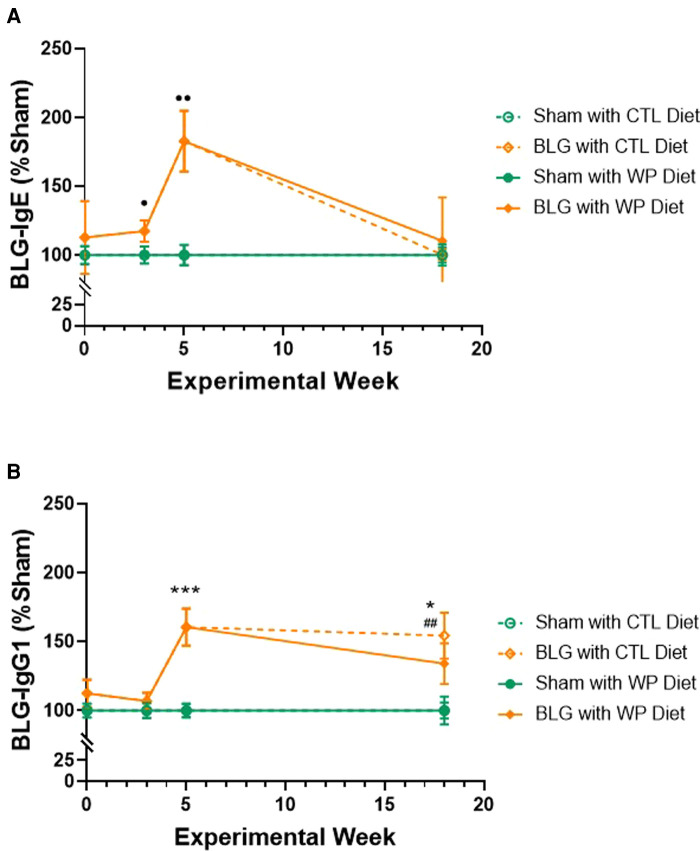
Relative quantification of BLG-specific immunoglobulin levels. Plasma samples were isolated from sham and BLG-sensitized mice on Weeks 0, 3, 5 and 18, and BLG-specific IgE (**A**) and IgG1 (**B**) were quantified using ELISA. The optical density (OD) value from each colorimetric reaction was normalized to the average OD value of the respective sham group and expressed as % sham. All mice were fed the CTL diet during the sensitization period, and thus, the mice receiving the same sensitization were grouped for Week 0 (*n* = 15–19), Week 3 (*n* = 23), and Week 5 (*n* = 24). For Week 18, the values from the four experimental groups are individually indicated (*n* = 12 per group). Sham *vs*. BLG groups at Week 0, 3, and 5 were compared using Mann–Whitney’s *U*-test; the four experimental groups at Week 18 were compared using two-way ANOVA. Values shown are the group average ± SEM. *p* < 0.05, *p* < 0.01 (Mann–Whitney’s *U*-test); ***p < 0.001, *p < 0.05 (two-way ANOVA with Tukey *post hoc* test); *p* < 0.01 (two-way ANOVA with Fisher’s LSD). See Supplementary Figure S1 for detailed values at the individual time points.

We first assessed the effect of long-term dietary allergen consumption on hypersensitivity immune responses in this mouse model of CMA by quantifying allergen-specific immunoglobulin levels in sham and BLG-sensitized mice after placing them on either the WP or CTL diet for 3 months. Blood samples were taken before, during, and after the sensitization phase (Week 0, 3, and 5, respectively) and at the end of the 3-month WP diet. The levels of BLG-specific IgE and IgG1 were compared at each time point (Figure 2). The IgE level in the sensitized group showed a gradual increase during the sensitization phase, starting to become significantly elevated compared to the sham mice after 3 weekly sensitization regimens (Figure 2A, sham: 100 ± 6%, *n* = 23; BLG: 117 ± 8%, *n* = 23; *p* = 0.0408, Mann-Whitney’s *U*-test). By the time the 5-week sensitization phase was completed, the levels of allergen-specific IgE had risen approximately 2-fold in the sensitized mice compared to sham mice (sham: 100 ± 7%, *n* = 24; BLG: 183 ± 22%, *n* = 24; *p* = 0.0012, Mann–Whitney’s *U*-test). After the subsequent exposure, the elevated IgE in BLG-sensitized mice declined to the sham levels, regardless of the diet type they consumed. However, it should be noted that large variations in individual IgE responses were observed in BLG-sensitized mice, as we previously reported (11, 23). In some of the sensitized mice that consumed the WP diet, allergen-specific IgE levels continued to rise after the sensitization and remained elevated at the time of sacrifice. Detailed data of IgE levels with individual values are presented in Supplementary Figure S1.

The allergen-specific IgG1 also rose in the sensitized group after 5 weeks (Figure 2B), showing a 1.6-fold greater level than the sham group (sham: 100 ± 4.9%, *n* = 24; BLG: 160.5 ± 13.5%, *n* = 24; *p* = 0.0002, Mann–Whitney’s *U*-test). By the end of the exposure phase, the elevated IgG1 levels in the BLG-sensitized group with the WP diet showed a decline to 1.3-fold of the respective sham group (sham/WP: 100 ± 10%, *n* = 12; BLG/WP: 134 ± 15%, *n* = 12; *p* = 0.24, two-way ANOVA). However, the level of BLG-specific IgG1 in the sensitized group with the CTL diet remained elevated until the end of the exposure phase (sham/CTL: 100 ± 6%; BLG/CTL: 154 ± 17%; *p* = 0.0197, two-way ANOVA). Detailed data of IgG1 levels with individual values are presented in Supplementary Figure S2.

These immunoglobulin analyses indicated that continued consumption of the offending allergen reduced allergen-specific IgE as effectively as the avoidance of the allergen in this mouse model of CMA. In contrast, the elevated level of IgG1 in BLG-sensitized mice was sustained with the CTL diet while the WP diet reduced it slightly, although the difference between the two groups was not statistically significant at this time point.

### BLG-sensitized mice exhibited behavioral changes with distinct manifestations that depended on allergen exposure

**Figure 3:**
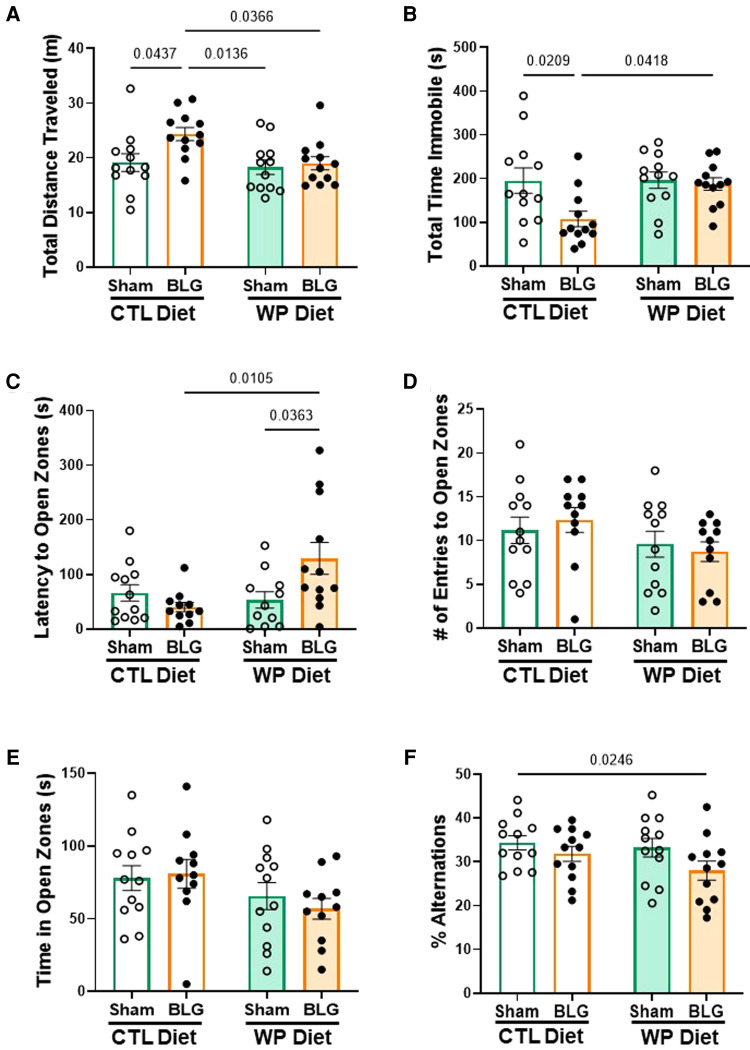
Behavioral assessments. All mice were subjected to behavioral tests during the last week of the 3-month exposure phase. Total distance traveled (**A**) and total time immobile (**B**) in the OFT, latency to enter the open zone (**C**), the number of entries to the open zone (**D**) and total time spent in the open zone (**E**) on the EZM, and the percentage of successful arm-entry alternations (% alternations) during the CMT (**F**) are compared among the experimental groups. Values indicate the group average ± SEM. Significant *p*-values are shown. Two-way ANOVA with Fisher’s LSD, *n* = 12 per group. Two outliers were identified by the ROUT (Q = 1%) for the latency to enter open zones and excluded from the final analysis (**C**). Two mice fell off the apparatus during the EZM test and thus were removed from the analysis of the number of entries to open zones (**D**).

During the last week of the 3-month allergen exposure phase, all mice underwent a series of behavior tests to assess sensitization- and/or diet-associated changes in their affective and cognitive behaviors. While the frequency and time spent in the center zone of the OFT were not significantly different among the groups (data not shown), BLG-sensitized mice that stayed on the CTL diet were more active than those placed on the WP diet and both of the sham groups, traveling longer distances (Figure 3A, sham/CTL: 19 ± 2 m, *n* = 12; BLG/CTL: 24 ± 1 m, *n* = 12; sham/WP: 18 ± 1 m, *n* = 12; BLG/WP: 19 ± 1 m, *n* = 12; sham/CTL *vs.* BLG/CTL *p* = 0.0437; BLG/CTL *vs*. sham/WP *p* = 0.0136 BLG/CTL *vs.* BLG/WP *p* = 0.0366, two-way ANOVA) and spending less time immobile (Figure 3B, sham/CTL: 196 ± 29 s, *n* = 12; BLG/CTL: 108 ± 18 s, *n* = 12; sham/WP: 196 ± 19 s, *n* = 12; BLG/WP: 187 ± 14 s, *n* = 12; sham/CTL *vs.* BLG/CTL *p* = 0.0209; BLG/CTL *vs.* BLG/WP *p* = 0.0418, two-way ANOVA) during the OFT. Conversely, sensitized mice that were continuously exposed to the allergen for 3 months exhibited increased latency to enter the open zones of the EZM (sham/CTL: 66 ± 15 s, *n* = 12; BLG/CTL: 41 ± 9 s, *n* = 11; sham/WP: 54 ± 15 s, *n* = 11; BLG/WP: 129 ± 29 s, *n* = 12; BLG/CTL *vs.* BLG/WP *p* = 0.0105; sham/WP *vs.* BLG/WP *p* = 0.0363, two-way ANOVA), where they would be vulnerable to a fall and exposed to a potential threat (Figure 3C). However, the number of entries to the open zones (Figure 3D) and the time mice spent in the open zones (Figure 3E) were not significantly different among the groups. In addition to their hesitance to explore the open zones of the EZM, BLG-sensitized mice on the WP diet also showed a tendency for declined spatial memory by performing fewer successful alternations during the CMT, although the difference did not reach statistical significance (Figure 3F, sham/WP: 33 ± 2%, *n* = 12; BLG/WP: 28 ± 2%, *n* = 12; *p* = 0.0607, two-way ANOVA, Fisher’s LSD). Sensitized mice on the CTL diet performed similarly to the sham group in this test (sham/CTL: 34 ± 2%, *n* = 12; BLG/CTL: 32 ± 2%, *n* = 12, *p* = 0.3612, two-way ANOVA, Fisher’s LSD). In all of the tests, the performances of the two sham groups were comparable to each other. These behavioral outcomes, assessed 3 months after the completion of sensitization and initiation of CTL or WP diet, may indicate that the activity levels of sensitized mice were influenced by the biological events triggered at the time of sensitization while the hesitancy to initiate their exploratory behavior was affected by the subsequent allergen exposure.

### Peripheral chemokine, CXCL13, was elevated in BLG sensitized mice after 3 months of allergen avoidance but not with continuous allergen exposure

As described in the previous section, BLG-sensitized mice exhibited small but significant behavioral changes after 3 months with or without continuous exposure to the allergen, although the levels of allergen-specific immunoglobulin levels had returned to the sham levels by this time. This observation led us to question whether other immune response mediators were present in BLG sensitized mice after the allergen exposure phase. As the mast cell activity indicator, systemic levels of MCPT-1 were quantified in the serum. Similarly, CXCL13 was also measured as a marker of follicular helper T cell (T_fh_) activity, plasma cell maturation, and antibody production (31, 32).

**Figure 4:**
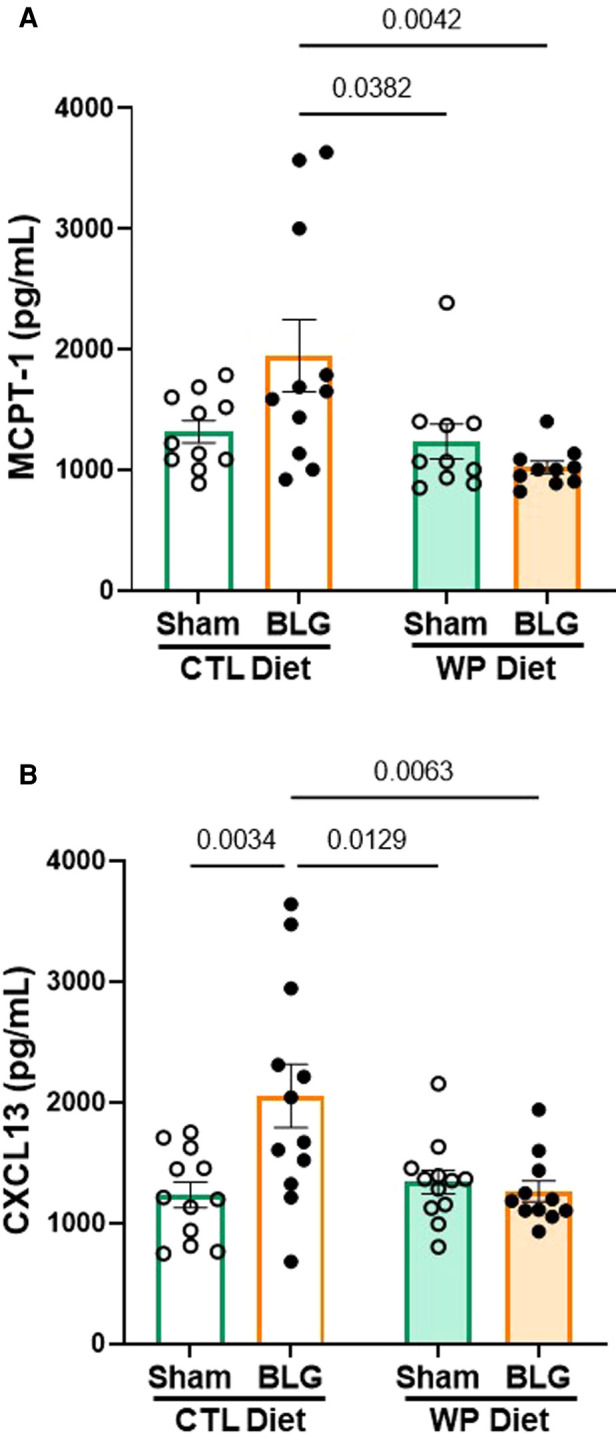
MCPT-1 and CXCL13 levels in the serum. The sera isolated from the terminal blood of sham and BLG-sensitized mice were used to quantify MCPT-1 (**A**) and CXCL13 (**B**). The concentrations of the analytes were determined from respective standard curves. Values indicate the group average ± SEM pg/ml. Significant *p*-values are shown. Two-way ANOVA, *n* = 10–12 per group. One outlier identified by the ROUT method (Q = 1%) was removed from the final analysis for MCPT-1 (**A**).

BLG-sensitized mice with CTL diet showed a trend for elevated serum MCPT-1 levels (Figure 4A), although the difference from the respective sham mice did not reach statistical significance (sham/CTL: 1,316 ± 92 pg/ml, *n* = 11; BLG/CTL: 1,947 ± 298 pg/ml, *n* = 11; *p* = 0.07, two-way ANOVA). However, the MCPT-1 levels in this group were significantly greater than the sham and sensitized mice that consumed the WP diet (sham/WP: 1,237 ± 144 pg/ml, *n* = 10, *p* = 0.0382; BLG/WP: 1,022 ± 52 pg/ml, *n* = 10, *p* = 0.0042, two-way ANOVA). The amount of CXCL13 in the sensitized mice with the CTL diet was significantly elevated compared to the rest of the groups (Figure 4B, sham/WP: 1,233 ± 106 pg/ml, *n* = 12; BLG/WP 2,053 ± 264 pg/ml, *n* = 12; sham/CTL: 1,339 ± 98 pg/ml, *n* = 12; BLG/WP: 1,264 ± 87 pg/ml, *n* = 11; comparison of BLG/CTL *vs.* sham/CTL: *p* = 0.0034, *vs.* sham/WP: *p* = 0.0129, *vs.* BLG/WP: *p* = 0.0063, two-way ANOVA). These observations suggested that sensitization-induced elevation of CXCL13 was not directly linked to the allergen-specific IgE levels after 3 months of allergen avoidance, whereas continuous allergen consumption lowered both the immunoglobulin and chemokine levels. Similarly, the dietary allergen seemed to also lower the overall sensitization-associated mast cell activity, decreasing the intra-group variability in MCPT-1 levels observed with allergen avoidance.

### Hippocampal astrogliosis was found in BLG-sensitized mice with both diets, while the extent of microgliosis might depend on the presence of dietary allergen

Because BLG-sensitized mice exhibited distinct diet-dependent behavioral changes after the exposure phase, we performed immunohistological analyses of their brains to examine neuroinflammation. Brain sections were stained for GFAP and Iba1 to identify astrocytes and microglia, respectively. The optical density of each immunostaining was subsequently quantified and compared among the experimental groups.

**Figure 5:**
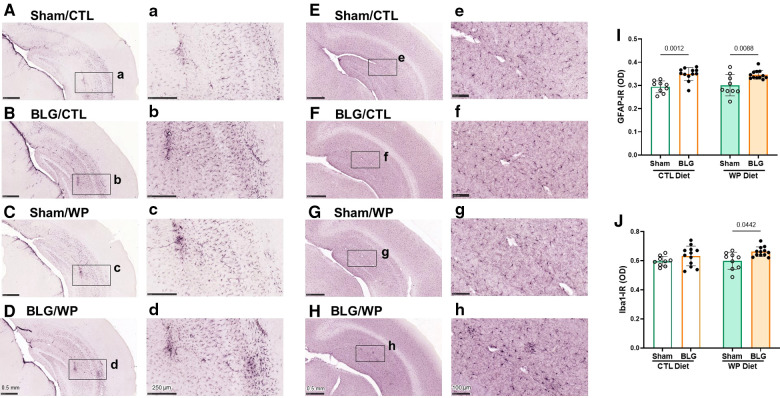
Immunohistochemical staining of GFAP and Iba1 in the brain. GFAP-positive astrocytes (**A–D**) and Iba1-positive microglia (**E–H**) were immunohistochemically identified in the brain sections from sham mice with the CTL diet (**A,E**), BLG mice with the CTL diet (**B,F**), sham mice with the WP diet (**C,G**), and BLG mice with the WP diet (**D,H**). The panels with lowercase letter labels in the right columns (**A–H**) depict high magnification images taken from the region indicated by the black rectangles in the corresponding photomacrographs (**A–H**). The low-magnification images were taken with 4X (**A–D**) or 4.5X (**E–H**) objectives, and the high-magnification images were taken with 14X or 20X objectives. Scale bars: 0.5 mm (**A–H**); 250 μm (**a–d**); 100 μm (**e–h**). The optical density (OD) of GFAP (**I**) and Iba1 (**J**) immunoreactivity (IR) within the hippocampal region was quantified using the QuPath image analysis software. Values indicate the group average ± SEM. Significant *p*-values are shown. Two-way ANOVA, *n* = 9–12 per group.

GFAP-positive cells were found throughout the brain, and they were particularly abundant in the hippocampus (Figures 5A–D). Overall, sham mouse brains showed relatively lower levels of GFAP staining (Figures 5A,C), although the staining appeared darker in the sham mice on the WP diet, particularly in perivascular regions (Figure 5C). On the other hand, the hippocampal GFAP staining in BLG-sensitized mice was more robust than in the respective sham groups, regardless of the diet they received during the exposure phase (Figures 5B,D). Again, strongly immunoreactive cells were clustered around the perivascular regions, exhibiting hypertrophic morphology.

In contrast, the differences in Iba1-positive microglia staining among the two sham groups and sensitized mice on the CTL diet were less apparent (Figures 5E–G). However, the microglia found in the hippocampus and cortical regions of BLG-sensitized mice on the WP diet showed less ramified, dense morphology, and some were found in cluster-like formations suggestive of microglia activation (Figure 5H).

Densitometric quantitation of the GFAP and Iba1 staining supported the qualitative observations. GFAP immunoreactivity (GFAP-IR) were significantly elevated in the brains of BLG-sensitized mice compared to their respective sham mice fed the same diet (Figure 5I, sham/CTL: 0.294 ± 0.008, *n* = 9; BLG/CTL: 0.349 ± 0.008, *n* = 12; sham/WP: 0.30 ± 0.02, *n* = 9; BLG/WP: 0.346 ± 0.006, *n* = 12; sham/CTL vs. BLG/CTL: *p* *=* 0.0012, sham/WP vs. BLG/WP: *p* *=* 0.0088, two-way ANOVA). However, the inter-group differences were rather small, and the sensitization-associated increases in the GFAP-IR were 19% and 15% for the CTL and WP diet groups, respectively. For Iba1 immunoreactivity (Iba1-IR), the difference between sham and BLG-sensitized mice were not observed with the CTL diet groups, but about 10% increase in the Iba1-IR was detected in the sensitized mice with the WP diet (Figure 5J, sham/CTL: 0.60 ± 0.01, *n* = 9; BLG/CTL: 0.63 ± 0.02, *n* = 12; sham/WP: 0.60 ± 0.02, *n* = 9; BLG/WP: 0.66 ± 0.01, *n* = 12; sham/CTL vs. BLG/CTL: *p* *=* 0.4151, sham/WP vs. BLG/WP: *p* *=* 0.0442, two-way ANOVA). These results indicated that astrogliosis developed in BLG-sensitized mice regardless of the diet, although the extent of microglial activation seemed to depend on continued exposure to the dietary allergen.

### CD45-immunoreactive leukocytes were largely localized in the meningeal and ependymal tissues and more frequently found in BLG-sensitized mice

**Figure 6:**
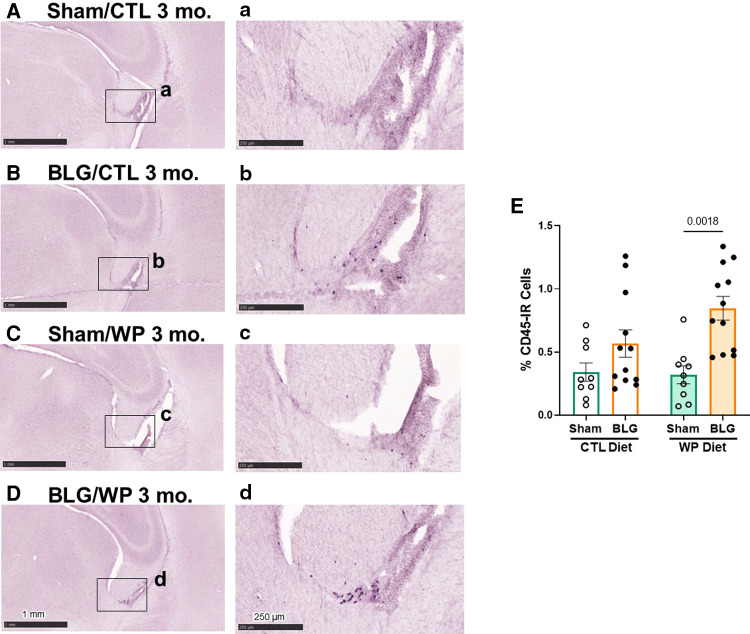
CD45-immunoreactive leukocytes in the brain. Brain sections from the experimental mice were immunostained for CD45. Representative staining from sham mice with the CTL diet (**A**), BLG mice with the CTL diet (**B**), sham mice with the WP diet (**C**), and BLG mice with the WP diet (**D**) are shown. Images of the fimbria within the lateral ventricles (**A–D**) were taken at higher magnification from the region indicated by the black rectangles in the corresponding photomacrographs (**A–D**). The images were taken with 1.5X (**A–D**) or 10X (**a–d**) objectives. Scale bars: 1 mm (**A–D**); 250 μm (**a–d**). The percentages of CD45-IR cells within the fimbria region were quantified using the QuPath image analysis software (**E**). Values indicate the group average ± SEM. Significant *p*-values are shown. Two-way ANOVA, *n* = 9–12 per group.

We postulated that sensitization-stimulated immune cells might have played a role in the activation of astrocytes in the BLG-sensitized groups (Figures 5A–D, I) and microglia in sensitized mice on the WP diet (Figures 5E–H, J). Thus, we histologically examined the presence of the CD45-immunoreactive (CD45-IR) leukocyte population in the brain (Figure 6). CD45-IR cells were round or ovoid, and the majority were found in the leptomeninges, choroid plexus, and ependyma of the lateral ventricles. Some CD45-IR cells were also located sporadically in the parenchyma and on or near vascular walls throughout the brain, including but not limited to the olfactory bulbs, cerebral cortex, thalamus, striatum, hippocampus, and cerebellum (Supplementary Figure S3). The patterns of leukocyte distribution appeared consistent across all experimental groups, although the stained cells were more frequently found in the brain sections from the BLG-sensitized mouse groups, particularly in the fimbria of the hippocampus within the lateral ventricles (Figures 6B, D).

Because CD45-IR cells were most frequently observed near the fimbria region in the lateral ventricle, we next quantified CD45-IR in this region. Although CD45-IR cells in the brains of BLG-sensitized mice on the CTL diet showed an increasing trend, the difference from the respective sham group did not reach statistical significance (Figure 6E, sham/CTL: 0.34 ± 0.07%, *n* = 9; BLG/CTL: 0.6 ± 0.1%, *n* = 12, *p* *=* 0.3413, two-way ANOVA). However, with the WP diet, the difference in average CD45-IR between sham and BLG-sensitized groups was significant, elevated in the BLG/WP group by greater than 2.5 fold (Figure 6E, sham/WP: 0.32 ± 0.07%, *n* = 9; BLG/WP: 0.85 ± 0.09%, *n* = 12; *p* *=* 0.0018, two-way ANOVA). These observations indicated that leukocytes were present throughout the brain regardless of the sensitization status or diet type, but their recruitment to the central nervous system (CNS) were likely promoted in BLG-sensitized mice with allergen consumption, particularly in the ventricular, cisternal, and vascularized regions.

### Chemokine levels in the brains of sensitized mice were greater with continued allergen consumption

The serum analysis described above indicated that CXCL13 was significantly elevated in the circulation of BLG-sensitized mice with the CTL diet (Figure 4B). Since CXCL13 has been reported to facilitate lymphocyte recruitment to the CNS (33) and increased levels of CXCL13 have been found in various neurological disorders (34, 35), we investigated the level of the chemokine in our mice. In addition, based on an initial screening of hippocampal lysates with an ELISA-based array, we also examined the levels of CCL12, or monocyte chemoattractant protein 5 (MCP-5), known to be involved in immune responses associated with allergic inflammation (36).

**Figure 7:**
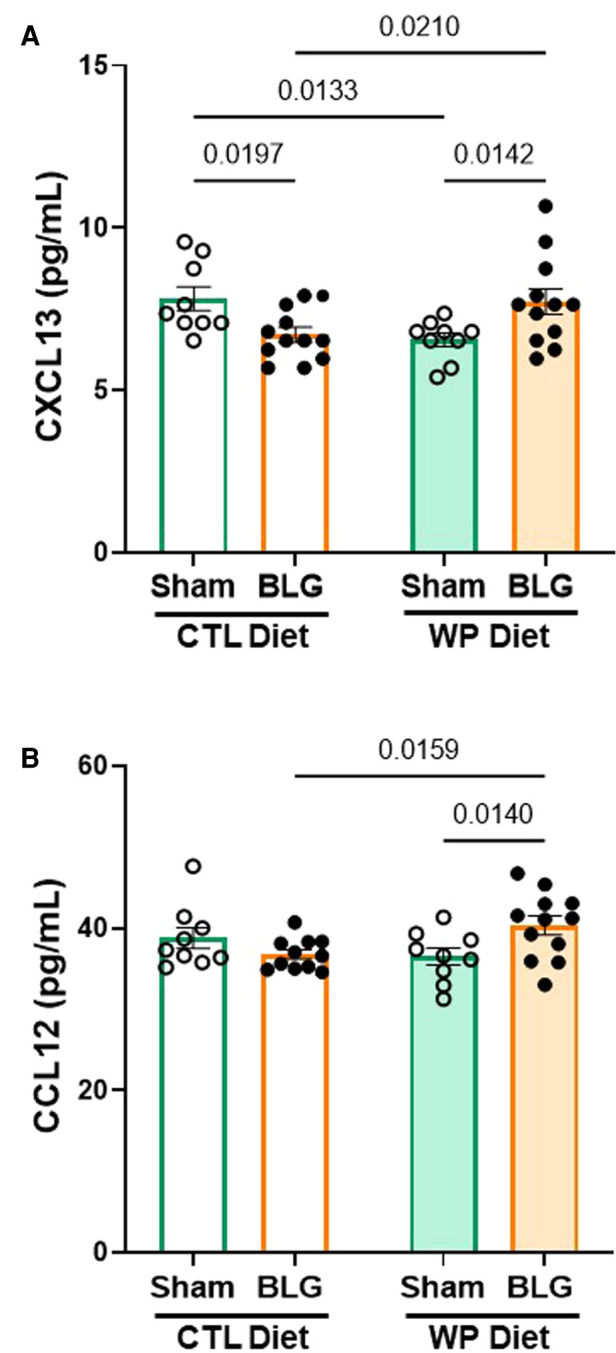
Chemokine levels in the hippocampal region. The hippocampus dissected from the right brain hemisphere of each mouse was lysed to extract total proteins. Ten µg of the hippocampal lysates were used to quantify CXCL13 (**A**) and CCL12 (**B**) using ELISA. The concentrations of the analytes were determined from respective standard curves. Values indicate the group average ± SEM. Significant *p*-values are shown. Two-way ANOVA, *n* = 9–12 per group.

Unlike in the serum samples, CXCL13 was not elevated but was slightly reduced in the hippocampal lysates from BLG-sensitized mice with the CTL diet compared to the respective sham group (Figure 7A, sham/CTL: 7.8 ± 0.4 pg/ml, *n* = 9; BLG/CTL: 6.7 ± 0.2 pg/ml, *n* = 12; *p* = 0.0197, two-way ANOVA). On the other hand, the sensitized mice with the WP diet showed small but significant elevation in the chemokine level, by ∼18% compared to their respective sham group and ∼18% compared to the sensitized group with the CTL diet (sham/WP: 6.5 ± 0.2 pg/ml, *n* = 9; BLG/WP: 7.7 ± 0.4 pg/ml, *n* = 12; BLG/CTL *vs.* BLG/WP *p* = 0.0210; sham/WP *vs.* BLG/WP *p* = 0.0142, two-way ANOVA). Similarly, a slight but significant elevation of CCL12 was detected in the WP-diet-fed BLG-sensitized mice compared to the respective sham group (8%) or the CTL-diet-fed BLG-sensitized group (10%) (Figure 7B, sham/CTL: 39 ± 1 pg/ml, *n* = 9; BLG/CTL: 36.8 ± 0.6 pg/ml, *n* = 11; sham/WP: 37 ± 1 pg/ml, *n* = 9; BLG/WP: 40 ± 1 pg/ml, *n* = 12; BLG/CTL *vs.* BLG/WP *p* = 0.0159; sham/WP *vs.* BLG/WP *p* = 0.0140, two-way ANOVA with Fisher’s LSD). Our data indicated that the hippocampal tissues from the BLG-sensitized mice with the WP diet contained greater amounts of these chemokines, although the difference from the sham mice was marginal. While these results are suggestive of increased CNS recruitment of leukocytes, further investigation is necessary to determine whether the limited changes in the analytes can be explained by cell type- or subregion-specific production of the chemokines or their diffusion into the cerebrospinal fluid.

### The presence of dietary WP increased intestinal leukocyte density in the ileum

**Figure 8:**
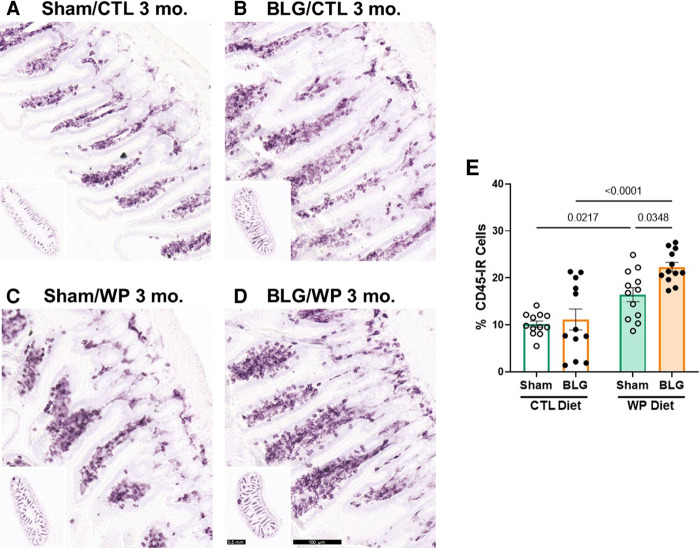
CD45-immunoreactive leukocytes in the intestine. Carnoy-fixed paraffin-embedded intestines were sectioned and stained for CD45 immunoreactivity. Ileal sections from sham mice with the CTL diet (**A**); BLG mice with the CTL diet (**B**); sham mice with the WP diet (**C**), and BLG mice with the WP diet (**D**) are shown. The lower left inset in each panel shows the representative cross-section of the group at a lower magnification. Scale bars: 100 μm (**A–D**); 0.5 mm (insets). The percentages of CD45-IR cells within ileal cross-sections were quantified from three sections for each animal using the QuPath image analysis software (**E**). Values indicate the group average ± SEM. Significant *p*-values are shown. Two-way ANOVA, *n* = 12 per group.

Since the intestine is the site of food allergen entry for oral sensitization and exposure, we also investigated immune cell activities in the intestines. For this purpose, paraffin-embedded ileal sections were stained for CD45-IR leukocytes (Figure 8). The differences in the overall staining intensities were evaluated by a pathology resident (YW), who was blinded to the identities and experimental conditions of the mice from which the intestinal sections originated. The experimental groups were then ranked in the order of low to high staining intensities as follows: sham/CTL ≤ BLG/CTL < sham/WP ≤ BLG/WP (Figure 8A–D).

This qualitative comparison was largely supported by the densitometric quantitation of the staining. Overall, the mice that received the WP diet showed more robust CD45-IR in their ileums (Figure 8E). While some of the BLG-sensitized mice with the CTL diet showed elevated levels of CD45-IR, the staining intensity in this group varied widely, and the difference from the respective sham group was not significant (sham/CTL: 10 ± 2%, *n* = 12; BLG/CTL: 11 ± 8%, *n* = 12, *p* = 0.9641, two-way ANOVA). On the other hand, CD45-IR in WP-diet-fed BLG-sensitized group was significantly greater than their sham mice with a 37.5% increase (Figure 8E, sham/WP: 16 ± 5%, *n* = 12; BLG/WP: 22 ± 3%, *n* = 12; sham/WP *vs.* BLG/WP *p* = 0.0348, two-way ANOVA), and 2-fold greater than the BLG/CTL group (*p* < 0.0001, two-way ANOVA). Furthermore, as noted by our qualitative observation of the staining, intestinal CD45-IR in the sham/WP group was greater than sham/CTL group (*p* = 0.0217).

These results suggested that the consumption of dietary whey resulted in increased trafficking of CD45-IR immune cells in the intestine regardless of the sensitization status, and allergen sensitization further facilitated such dietary-allergen-induced leukocyte recruitment. Phenotyping these leukocytes will clarify the cell types present and their diet- and sensitization-specific roles at the site of allergen entry.

## Discussion

Both clinical and preclinical studies have contributed to our current understanding of food allergy, particularly elucidating the mechanisms of hypersensitivity development and efficacies of therapeutic approaches for severe immediate reactions. However, some individuals are clinically known to be asymptomatically sensitized, having elevated allergen-specific immunoglobulin levels but able to tolerate the consumption of the offending allergen without life-threatening responses (18, 19). We questioned whether continued consumption of offending allergen by asymptomatically sensitized individuals would result in long-term inflammation and associated pathologies. In the present study, we addressed this question using the mouse model of non-anaphylactic CMA, previously shown to produce significantly elevated levels of allergen-specific IgE in C57BL/6J mice without severe immediate reactions upon allergen challenge (11, 23). Long-term consumption of dietary allergen was simulated by placing BLG-sensitized mice on the WP diet for 3 months, whereas successful avoidance of offending allergen was modeled by maintaining a separate set of sham and BLG-sensitized mice on the whey-free CTL diet.

Upon hypersensitivity assessment by allergen-specific immunoglobulin levels, we found that the allergen-specific IgE levels were reduced in sensitized mice regardless of the diet type they consumed during the exposure phase (Figure 2A). In the sensitized group with the CTL diet, the decline in the IgE might have resulted from reduced production of the antibody. The relatively short half-life of unbound IgE (37–39) could then lead to eventual elimination from the circulation. Alternatively, allergen-specific IgE produced during the sensitization phase might have become rapidly associated with the high-affinity Fc*ε* receptor I (Fc*ε*RI) on the surface of mast cells and basophils, reducing the levels of free IgE detected by the assay. These possibilities may be tested by evaluating the IgE load on the granulocyte populations or quantifying Fc*ε*RI, which is stabilized by the association with IgE (40). Indeed, we have recently demonstrated that the dural tissue from BLG-sensitized mice contained IgE-IR cells after 1 month of consuming the WP diet, whereas no IgE-IR cells were found in their respective sham group (25). On the other hand, the declined levels of BLG-specific IgE in the WP diet group may indicate allergen-induced desensitization. Indeed, daily administration of offending allergens in tolerated dosages is the fundamental practice of oral immunotherapy (OIT) to facilitate desensitization (41).

In contrast to IgE, the levels of BLG-specific IgG1 remained relatively elevated 3 months after the sensitization phase, especially with the CTL diet group (Figure 2B). The IgG1 levels declined slightly in the WP diet group, suggesting the desensitization process in this group. With the half-life of IgG1 being 6–8 days compared to the 12 h of IgE in their unbound forms (38), it was likely that BLG-specific IgG1 remained in the circulation longer than IgE. Nonetheless, an additional detailed assessment with more time points is required to fully understand the dynamics of shifts in immunoglobulin levels during the allergen exposure phase in this model.

We also performed behavioral tests to assess the possible association of the changes in the plasma immunoglobulin levels with the manifestation of CMA-associated behavioral symptoms. We previously reported that BLG-sensitized mice exhibited anxiety- and depression-like behavior shortly after an acute allergen challenge, even in the absence of immediate anaphylactic response (11, 23). Sensitized mice showed behavioral changes, as we observed with acutely challenged CMA mice. However, the behavioral phenotype was distinct between the two diet groups (Figure 3) and from the acute-challenge experiments mentioned above. While the CTL diet increased the overall activity of sensitized mice (Figures 3A,B), the 3-month consumption of the WP diet led to anxiety-like behavior (Figure 3C). The BLG/WP diet group also showed the tendency towards spatial memory impairment (Figure 3F), suggesting that dietary allergen could elicit mild cognitive decline. Although the specific effects of the allergen on behavior need to be further examined with varying amounts of the allergen and the frequencies and durations of exposure, these behavioral observations led to the possibility that asymptomatically sensitized individuals may experience different types of behavioral manifestations depending on whether they avoid or consume the offending allergen.

Without a well-defined association between the systemic immunoglobulin levels and behavioral changes, we sought for other immunologic factors present in the circulation after the allergen avoidance/exposure phase (Figure 4). As the markers of mast cell and T_fh_ cell activities, serum levels of MCPT-1 and CXCL13 were measured, respectively. MCPT-1 is one of many preformed granule contents mucosal mast cells release upon activation (39), and its serum level is elevated in a mouse model of CMA with increased repetitive behavior (42). The quantification of MCPT-1 values showed an increasing trend in the BLG/CTL diet group, although considerable individual variability existed in this group and the comparison of the group average values with the respective sham group did not reach statistical significance. However, in some of the animals within the group showed greatly elevated MCPT-1 levels, indicating that the activity of mast cells, likely intestinal mucosal mast cells, was elevated without additional allergen exposure (Figure 4A). While the difference between these mice and the rest of the mice within the group is unclear, it is of our interest to further examine the differential immune responses in these subgroups. Nonetheless, with MCPT-1 level the BLG/CTL diet group having an increasing trend similar to the overall activity measured during the OFT (Figures 3A,B), it is attractive to postulate the involvement of mast cell overactivity in the induction of motor hyperactivity, as discussed by a recent review on the role of intestinal mast cells in neuroinflammation and the pathogenesis of attention deficit hyperactivity disorder (43).

CXCL13 has been well-characterized to promote the chemotaxis of lymphocytes expressing its receptor, CXCR5, to secondary lymphoid organs (44). Interestingly, it has been reported that CXCL13 facilitates the accumulation of differentiated B lymphocytes in the CNS (45). Furthermore, elevated levels of CXCL13 have been reported in the cerebrospinal fluid (CSF), active plaques, and meningeal ectopic follicles of patients with multiple sclerosis (MS), and thus, is considered to be a biomarker for MS and other neuroinflammatory diseases (33, 34, 46). Because this chemokine was also elevated in the brains of our sensitized mice with the WP diet (Figure 7A), it could be postulated that BLG sensitization triggered the recruitment of lymphocytes to the CNS, likely a subset of differentiated B cells, maintained by the continuous allergen exposure. The increase in CXCL13 level of the BLG/CTL group was even more pronounced (Figure 4B) than the increase in MCPT-1 level in this group, while neither of these analytes was elevated in the BLG/WP diet group. These results may signify that persisting immunological changes occurred during sensitization, leading to increased mast cell activity and chemokine release in the BLG/CTL diet group. In contrast, continuous consumption of the allergen seemed to have lessened the sensitization-induced peripheral immune responses but not neuroinflammation (Figures 5–7).

This notion was supported by the observation that CD45-IR cells were more frequently found in the brains of sensitized mice, particularly with the WP diet, than sham mice (Figure 6, Supplementary Figure S3). Interestingly, similar patterns of leukocyte infiltration in the CNS have been reported in a study using rodents with adjuvant-induced peripheral inflammation and experimental autoimmune encephalomyelitis (EAE), a mouse model of MS (47). The thorough investigations of the phenotypes and anatomical locations of leukocytes in the brain revealed that the choroid plexus and extraventricular vessels provided the routes of leukocyte infiltration *via* the CSF, with the fimbria being one of the commonly infiltrated areas as we observed in our sensitized mice (Figure 6).

Although our results suggested that leukocyte trafficking to the CNS increased in sensitized mice, the differences among the experimental groups should be further validated in the future by quantifying the cells in specific brain regions, meninges, and CSF. In addition, the phenotypes of these infiltrated leukocytes need to be identified to determine their specific roles. Based on the microglia phenotype in the brains of sensitized mice with the WP diet (Figure 5H), the infiltrated leukocytes likely served as mediators that communicated food-allergen-induced peripheral inflammation to the CNS and activated microglia, influencing the behavior of the mice *via* neuroinflammation.

Unlike the Iba1 staining, GFAP-IR astrocytes were abundant in the sensitized mice of both diet groups (Figures 5B,D). Hypertrophic perivascular astrocytes were also found in our previous study with the CMA mouse model after acute allergen challenge (23). The presence of astrogliosis at 1 week (acute challenge model) and 3 months (chronic exposure model) after the completion of the sensitization regimen has suggested that astrocytes become activated during the sensitization phase and remain reactive for prolonged periods. It is also noteworthy that the sham mice with the WP diet showed more hypertrophic perivascular astrocytes in the brain (Figures 5A,C) and CD45-IR cells in the ileum (Figures 8A,C) than the sham mice that never received WP. Introducing the novel allergenic dietary protein may initially trigger a subtle level of immune responses in the CNS, as well as in the intestines.

It should be noted that the quantified differences in the cytokine levels and gliosis in the brain with sensitization and/or continued allergen exposure were rather modest. These small but significant differences may signify localized, yet consistent changes with the experimental groups. For example, hypertrophic astrocytes were mainly found near larger vessels, and CD45-IR cells were more frequently found in the fimbria region of the hippocampus. Assessing these parameters from regions of interest could dilute cell-specific events within the regions. Whether or how these highly specific changes directly or indirectly affect behavior is yet to be determined in our future studies.

How do these findings in our animal model affect the concept of OIT, in which increasing amounts of offending allergens are continuously ingested by food-allergic patients? While our results suggested that continuous allergen exposure could result in persistent neuroinflammation, we cannot assume that our observations in mice with experimentally induced allergic sensitization directly apply to human patients with idiopathic food allergies. OIT aims to develop immune tolerance in severely symptomatic patients to prevent life-threatening reactions upon accidental exposure. The immunological process of tolerance established by this methodology is also likely distinct from that of tolerance in asymptomatically sensitized individuals. Importantly, the benefit of OIT to prevent deadly reactions outweighs the potential risk of developing neurological diseases later in life. Thus, additional in-depth investigations for different types of allergic patients are necessary before any clinical recommendation may be proposed.

## Conclusion

We demonstrated that continuous allergen consumption by asymptomatically sensitized CMA mice resulted in persisting neuroinflammation and behavioral changes despite decreased levels of allergen-specific IgE in the circulation. It may be unlikely for sensitized individuals to include allergens intentionally and consistently in their diets as we treated our BLG-sensitized mice during the exposure phase. However, as the implementation of OIT increases as a treatment option for food allergies in patients who are able to tolerate the allergens (41, 48), neuroinflammation and associated disorders may be potential long-term risks to be considered.

## Data Availability

The original contributions presented in the study are included in the article/**Supplementary Material**, further inquiries can be directed to the corresponding author/s.
