# Supplementary figures and images for: Asymptomatic sensitization to a cow’s milk protein induces sustained neuroinflammation and behavioral changes with chronic allergen exposure
Source: Front Allergy. 2022 Sep 7;3:870628. doi: 10.3389/falgy.2022.870628 (PMC9490182; doi:10.3389/falgy.2022.870628)

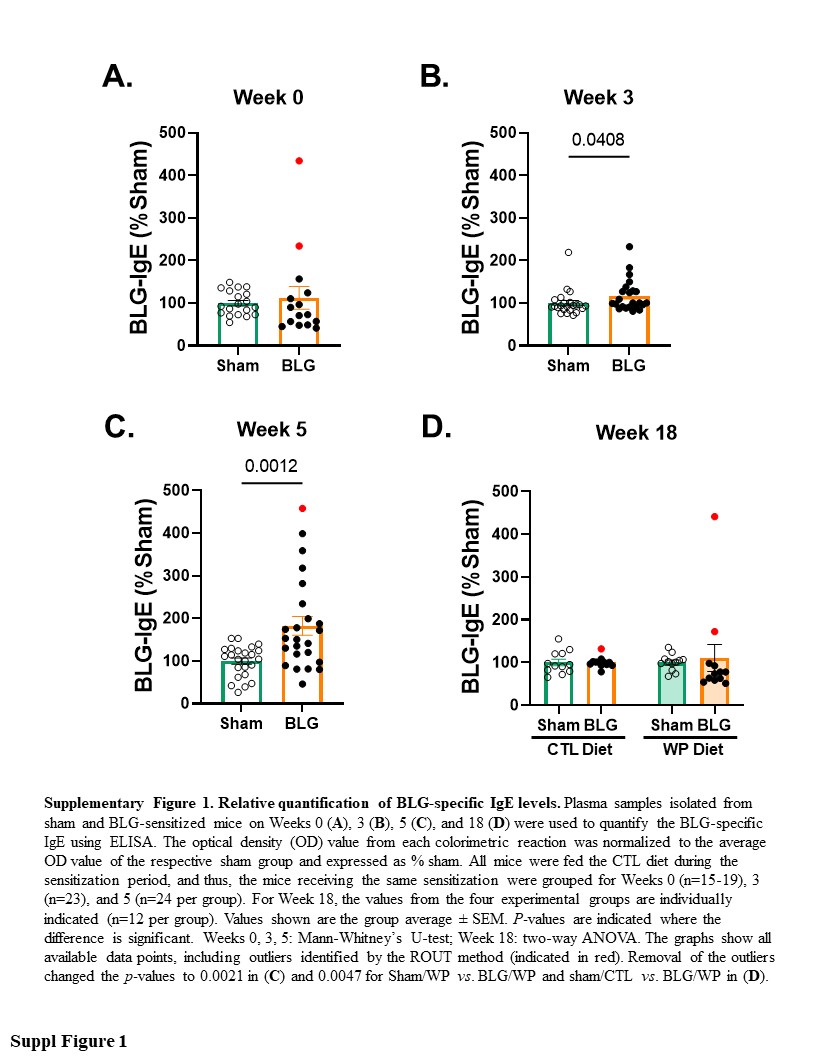

Supplement: Supplementary file 1 [file Image_1_v1.jpeg]

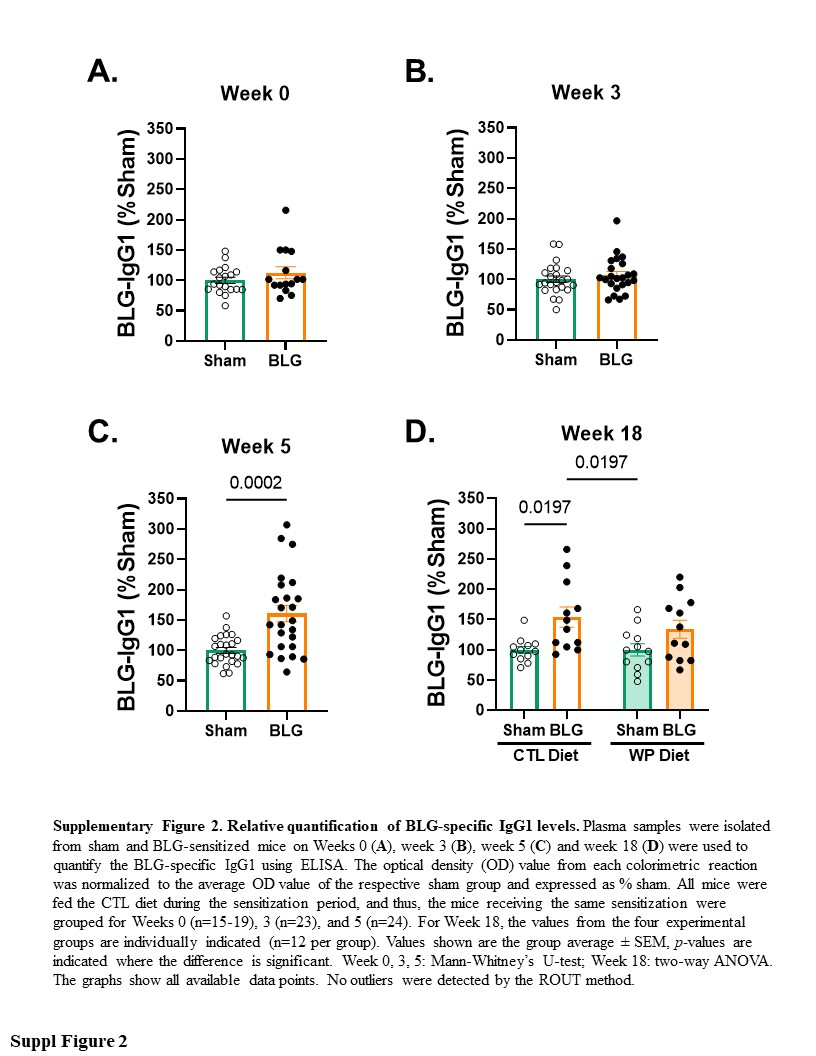

Supplement: Supplementary file 2 [file Image_2_v1.jpeg]

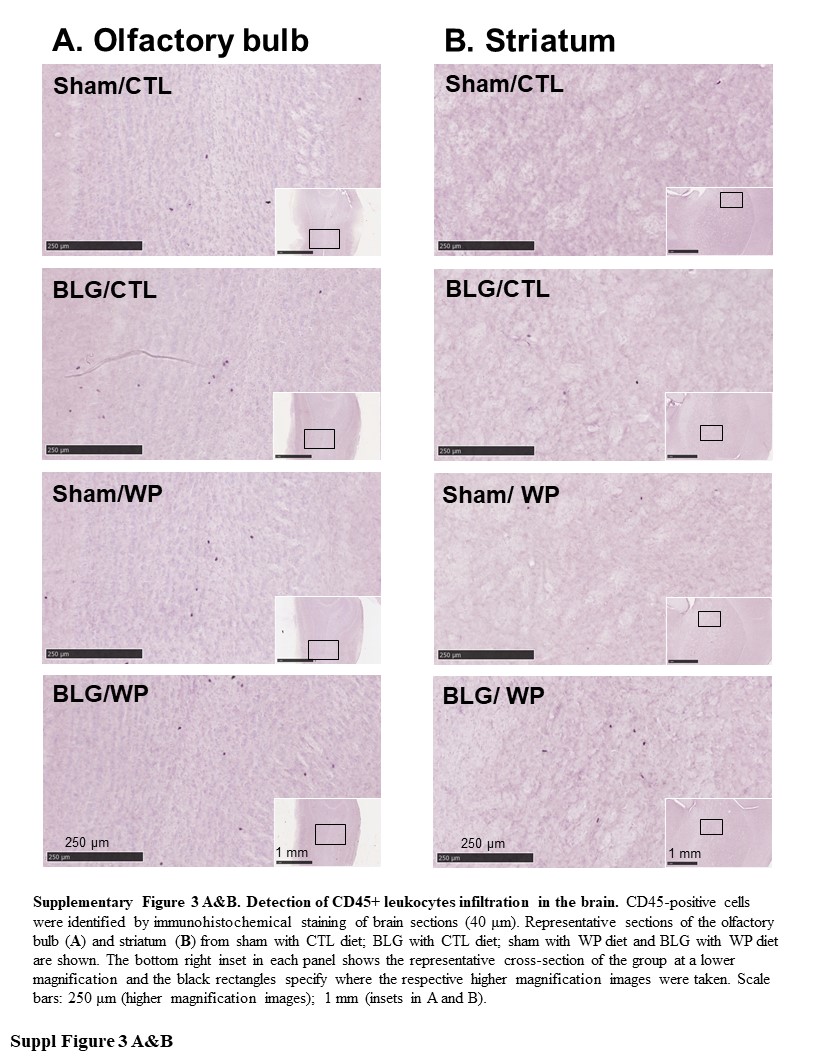

Supplement: Supplementary file 3 [file Image_3_v1.jpeg]

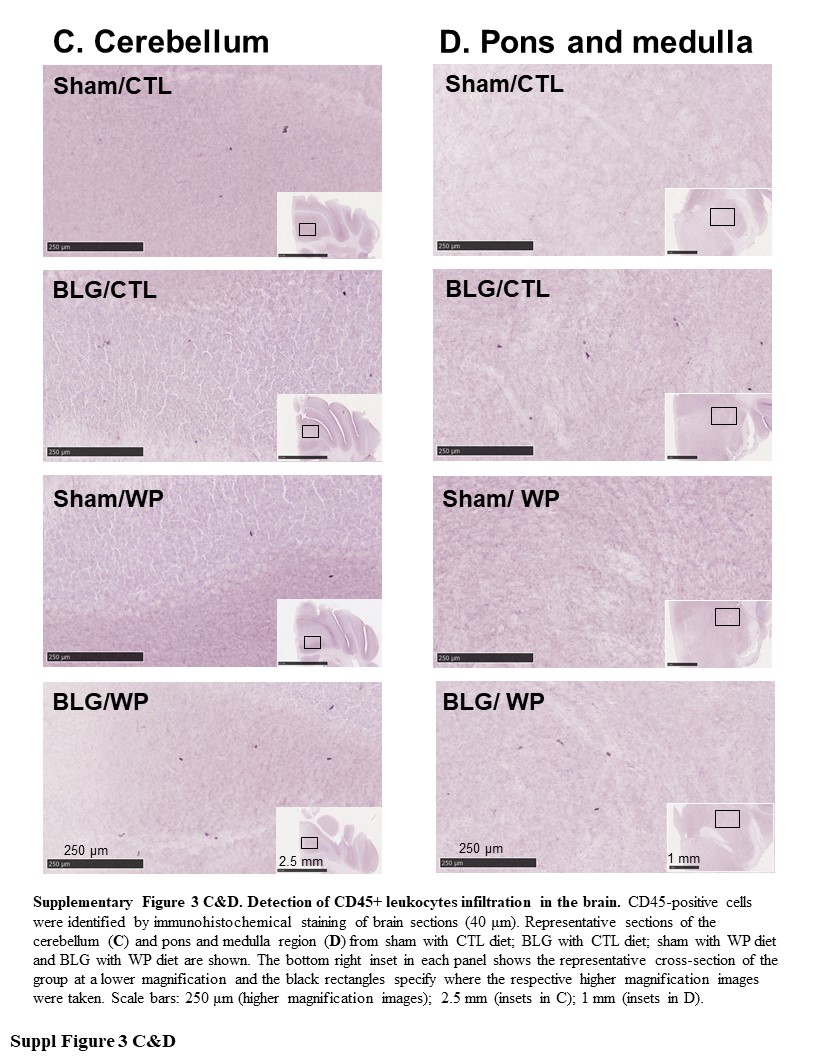

Supplement: Supplementary file 4 [file Image_4_v1.jpeg]
